# P58 From awareness to action: bridging the knowledge – practice gap in antimicrobial resistance in Nigeria—a systematic review of community-based strategies

**DOI:** 10.1093/jacamr/dlag102.064

**Published:** 2026-06-26

**Authors:** Maureen Eloho Okorodudu, Charles Maibvise, Rasha Abdelsalam Elshenawy

**Affiliations:** School of Health, Medicine and Life Sciences, University of Hertfordshire, College Lane, Hatfield AL10 9AB, UK; School of Health, Medicine and Life Sciences, University of Hertfordshire, College Lane, Hatfield AL10 9AB, UK; School of Health, Medicine and Life Sciences, University of Hertfordshire, College Lane, Hatfield AL10 9AB, UK

## Abstract

**Background:**

Antimicrobial resistance (AMR) is a critical and escalating public health challenge in Nigeria, driven by inappropriate antibiotic use, weak regulatory enforcement, and limited diagnostic capacity.^1^ National and global frameworks, including Nigeria’s National Action Plan (NAP II) and the WHO Global Action Plan, emphasize awareness and community engagement as key strategies. However, the extent to which awareness translates into appropriate antibiotic use remains unclear.^2^

**Objectives:**

To evaluate the effectiveness of community-based AMR awareness strategies in Nigeria, to examine the relationship between knowledge and antibiotic use behaviour, and to generate evidence-based recommendations to support progress towards NAP II and WHO Global Action Plan targets.

**Methods:**

A systematic review was conducted following PRISMA 2020 guidelines. PubMed, Scopus and African Journals Online were searched for English-language studies published between 2015 and 2025. The PEO framework guided the search strategy, focusing on Nigerian populations, community-based AMR awareness strategies and related outcomes. Selected articles were critically appraised using CASP tools. Data extraction captured study characteristics, AMR awareness levels, knowledge–practice outcomes and intervention strategies. Ethical approval was not required as no patient-identifiable data were used.

**Results:**

From 363 identified records, 14 studies were ultimately included in the final synthesis, 13 cross-sectional surveys and one multi-phase modelling study. AMR awareness varied widely across populations, from 17% demonstrating good knowledge among adult outpatients to 94.9% among patent medicine vendors, reflecting marked inconsistency in awareness levels. A consistent knowledge–practice gap appeared across 10 of the 14 studies. For example, while 94.9% of patent medicine vendors understood AMR causes, 59.9% continued dispensing antibiotics without prescription. Similarly, although 82.7% of physicians demonstrated good AMR knowledge, 67.2% reported routine empirical prescribing without diagnostic confirmation, and 75.7% of healthcare workers prescribed antibiotics inappropriately for sore throats. Most studies were descriptive in design, providing limited evidence on intervention effectiveness. Two studies involving structured educational programmes for medical and healthcare students improved knowledge scores (64.7% and 57.1% good knowledge, respectively) but showed limited impact on prescribing or dispensing behaviour. The strongest evidence for impact was a multi-component modelling study, which projected that combining public education, antimicrobial stewardship and over-the-counter antibiotic sales regulation could reduce Nigeria’s AMR burden by up to 55% over ten years.

**Conclusions:**

This review demonstrates that while AMR awareness exists across multiple populations in Nigeria, it does not consistently translate into appropriate antibiotic use, highlighting a persistent and actionable knowledge–practice gap. The predominance of cross-sectional, descriptive studies limits understanding of interventions’ effectiveness in real-world settings, underscoring the need for rigorous evaluative research on multi-component, behaviour-focused interventions. Strengthening antimicrobial stewardship, improving regulatory enforcement of antibiotic dispensing, and integrating community-based education within health system frameworks are essential steps towards achieving NAP II and WHO Global Action Plan targets.
